# LAMP1/2 as potential diagnostic and prognostic marker for brain lower grade glioma: A review

**DOI:** 10.1097/MD.0000000000034604

**Published:** 2023-08-18

**Authors:** Xiao Fen Qiu, Xiaoli Chen

**Affiliations:** a Ganzhou People’s Hospital Central Laboratory, Ganzhou, China

**Keywords:** brain lower grade glioma, diagnostic marker, LAMP1, LAMP2, LAMPs, prognostic marker

## Abstract

Brain lower grade glioma (LGG) is a common type of glioma. The current treatment methods still have some limitations, and some LGG patients will inevitably continue to deteriorate after treatment. We found the value of lysosomal associated membrane proteins (LAMPs) in the diagnosis and prognosis of LGG, which helps to enhance the clinical understanding of LGG treatment and improved prognosis. We assess the role of LAMPs in LGG, via the publicly available TCGA database. We explored expression levels of LAMPs in LGG using GEPIA2, cBioPortal, and UALCAN databases. The correction of LAMPs expression levels with immune cell infiltration in LGG patient was assessed by TIMER database. The Lysosomal associated membrane protein 1 (LAMP1)/2/4 mRNA levels were significantly higher in LGG patients than in healthy controls. Morover, high mRNA expressions of LAMP1/2/Lysosomal associated membrane protein 3 were associated with poor overall survival. We found that the immune invasion of LGG was almost significantly correlated with the expression of LAMPs. The results suggested that mRNA expressions of LAMP1 and LAMP4 were significantly associated with histological subtypes in LGG patients. lysosomal associated membrane protein 2 and LAMP5 were significantly down-regulated expression in samples of TP53 mutant in LGG compared to TP53 wild type. In addition, Lysosomal associated membrane protein 3 and LAMP4 were significantly overexpressed in samples of TP53 mutant in LGG Enrichment analysis applied to each component indicated that biological function was primarily associated with series of pathways in synapse and immunity.

## 1. Introduction

Gliomas are the most common malignant primary brain tumors of the central nervous system in adults.^[1,2]^ The grade of a malignant tumor is usually determined by the rate at which the tumor cells spread or grow, and determines the prognosis, treatment, and management plan.^[3,4]^ According to the 2007 WHO Classification of Central nervous system tumors, grade I and grade II belong to brain lower grade glioma (LGG).^[5]^ At present, surgical resection of the tumor in combination with chemotherapy, radiotherapy and nerve repair therapy can currently improve patient outcomes, but more than 50% of LGG patients will eventually develop a highly aggressive glioma, which usually means poor treatment response.^[6,7]^ It is crucial to find effective biomarkers and new therapeutic targets for diagnosis or treatment and prognosis of LGG.

Lysosome is an important cytoplasmic organelle whose critical functions in cells are increasingly being understood. Lysosomal homeostasis has even been shown to play an active role in cancer biology.^[8]^ Lysosomes enhance the aggressiveness of cancer by releasing their enzymes during cell transformation and cancer progression.^[9]^ Lysosomal associated membrane proteins (LAMPs) are family of glycosylated proteins that exist mainly in the lysosomal membrane and are expressed differently in different tissues. LAMP proteins are involved in many different aspects of cell biology and can influence cellular processes such as phagocytosis, autophagy, lipid transport, and aging.^[10]^ Interestingly, for all the 5 members identified so far [lysosomal associated membrane protein 1 (LAMP1), lysosomal associated membrane protein 2 (LAMP2), lysosomal associated membrane protein 3 (LAMP3), lysosomal associated membrane protein 4, and Lysosomal associated membrane protein 5], a role in cancer has been suggested.^[11]^

LAMP1 and LAMP2 act as pre-invasion and pre-metastasis factors in cancer cells and are abnormally localized on the plasma membrane of cancer cells, such as human melanoma A2058 cells, human colon cancer CACO-2 cells, and human fibrosarcoma HT1080 cells.^[12]^ LAMP1 and LAMP2 can promote both the adhesion of cancer cells to extracellular matrix, basement membrane and endothelium and the migratory potential of cells during metastasis.^[13]^ In glioblastoma, pancreatic, and ovarian cancer, LAMP1 expression on the cell surface plays a role in the early stages of cancer progression, rather than during metastasis.^[14]^ In acute myeloid leukemia cells, LAMP1 knockout can reduce cancer cell viability by destroying lysosomes.^[15]^ LAMP2 is also highly expressed in poorly differentiated human gastric adenocarcinoma, hepatocellular carcinoma, salivary adenoid cystic carcinoma and in the broncho-alveolar lavage fluid of patients with lung adenocarcinoma, representing 1 novel molecular marker for these cancer types.^[16,17]^

In this study, we used databases including GEPIA2, cBioPortal, UALCAN to evaluate the expression levels and prognosis of LAMPs in disease and control in LGG, used TIMER database to detect the wettability levels of LAMPs. We have some evidence that helps us to understand more about the molecular characteristics of LGG.

## 2. Methods

### 2.1. GEPIA2 analyses

The GEPIA2 database^[18]^ (http://gepia2.cancer-pku.cn/index.html) is an interactive web server for analyzing the RNA sequencing expression data of 9736 tumors and 8587 normal samples from the TCGA and the GTEx projects, using a standard processing pipeline to address important questions in cancer biology. GEPIA2 provides customizable functions such as tumor/normal differential expression analysis, profiling according to cancer types or pathological stages, patient survival analysis, similar gene detection, correlation analysis and dimensionality reduction analysis. We employed the GEPIA2 Expression DIY module (default parameters) to analyzed mRNA expression of the LAMP gene family in LGG and normal tissues. Then survival map analysis was used to obtain heat maps of LAMP family gene survival in all cancers. Finally, we used the survival analysis module to assess relations between LAMP gene family gene expression and overall survival in LGG patients to plot survival curves. Hazar ratios were calculated based on the Cox PH (proportional hazard) model, the 95% confidence interval is indicated by dashed lines, the x-axis unit is months, and *P* < .05 is considered statistically significant.

### 2.2. UALCAN dataset analyses

UALCAN^[19]^ (http://ualcan.path.uab.edu) is an interactive web resource based on level 3 RNA-seq and clinical data of 31 cancer types from TCGA database. It can be used to analyze relative transcriptional expression of potential genes of interest between tumor and normal samples and association of the transcriptional expression with relative clinicopathologic parameters. In this study, UALCAN was used to analyze the mRNA expressions of 5 LAMPs family members in LGG tissues and their association with histological subtypes. Difference of transcriptional expression was compared by students test and *P* < .05 was considered as statically significant. TP53 mutation status is obtained from TCGA whole exome sequencing data. We downloaded Mutation Annotation Format files (derived from VarScan2) from Genomic Data Commons portal. The sample with/without TP53 mutation status were matched with RNA-seq data. In this study, UALCAN was used to analyze the mRNA expressions of 5 LAMPs family members in LGG tissues and their association with histological subtypes.

### 2.3. cBioPortal dataset analyses

The cBioPortal for cancer genomics (http://www.cbioportal.org) is an open-access repository of cancer genomics datasets.^[20,21]^ We investigated the copy number alteration and mutation landscape of LAMP gene family in pan cancer and LGG. We then employed cBioPortal to inspect the mutation frequency of LAMP gene family in the TCGA database (10,967 samples in 32 studies). We then employed cBioPortal to inspect the mutation frequency of LAMP gene family in LGG of the TCGA database (1693 samples in 3 studies).

### 2.4. Genome-wide correlation analyses

The Cancer Regulome Explorer (http://explorer.cancerregulome.org/) enables users to search, filter, and visualize analytical results generated from TCGA data and explore associations among heterogeneous features. We used it to display the expression of LAMPs family members and its correlation with other variables in LGG on the chromosomic level. Only associations with |pairwise correlation| ≥0.4 and -log_10_ (*P* value) ≥ 10 were shown in the circos plots.

### 2.5. Metascape analysis

Metascape (http://metascape.org) integrates more than 40 gene function annotation databases and supplies various visualization methods, allowing readily gene function analysis.^[22]^ Herein, we employed this database to perform enrichment analysis of LAMPs related genes obtained from the Cancer Regulome Explorer (http://explorer.cancerregulome.org/). The analysis included gene ontology and Kyoto Encyclopedia of Genes and Genomes enrichment analysis. We set min overlap as 3, min enrichment as 1.5, and *P* < .05 as significant.

### 2.6. Immune infiltration

TIMER is a comprehensive resource for systematical analysis of immune infiltrates across diverse cancer types.^[23]^ It provided the profile of 6 types of infiltrating immune cells (B cells, CD4^+^ T cells, CD8^+^ T cells, Neutrophils, Macrophages, and Dendritic cells) in tumor tissues which was detected by RNA-Seq expression profiling data. We assessed the correction of LAMPs expression levels with immune cell infiltration in LGG patient.

### 2.7. TIMER2.0 analysis

The TIMER2.0 database(http://timer.cistrome.org/) is a comprehensive resource for systematical analysis of immune infiltrates across diverse cancer types.^[24]^ Expression modules exploring associations between gene expression and tumor features in TCGA. We use the expression modules assessed the expression profiles of LAMP family genes in pan cancer (including LGG) samples with TP53 gene mutation and TP53 gene non-mutation. Heatmap shows the log_2_ fold changes of the differential expression of each gene for each cancer type. Five boxplots from heatmap to show the details in expression profiles of LAMP family genes in LGG samples with TP53 gene mutation and TP53 gene non-mutation.

## 3. Results

### 3.1. Genetic alteration of LAMP genes in LGG

**Figure 1.**
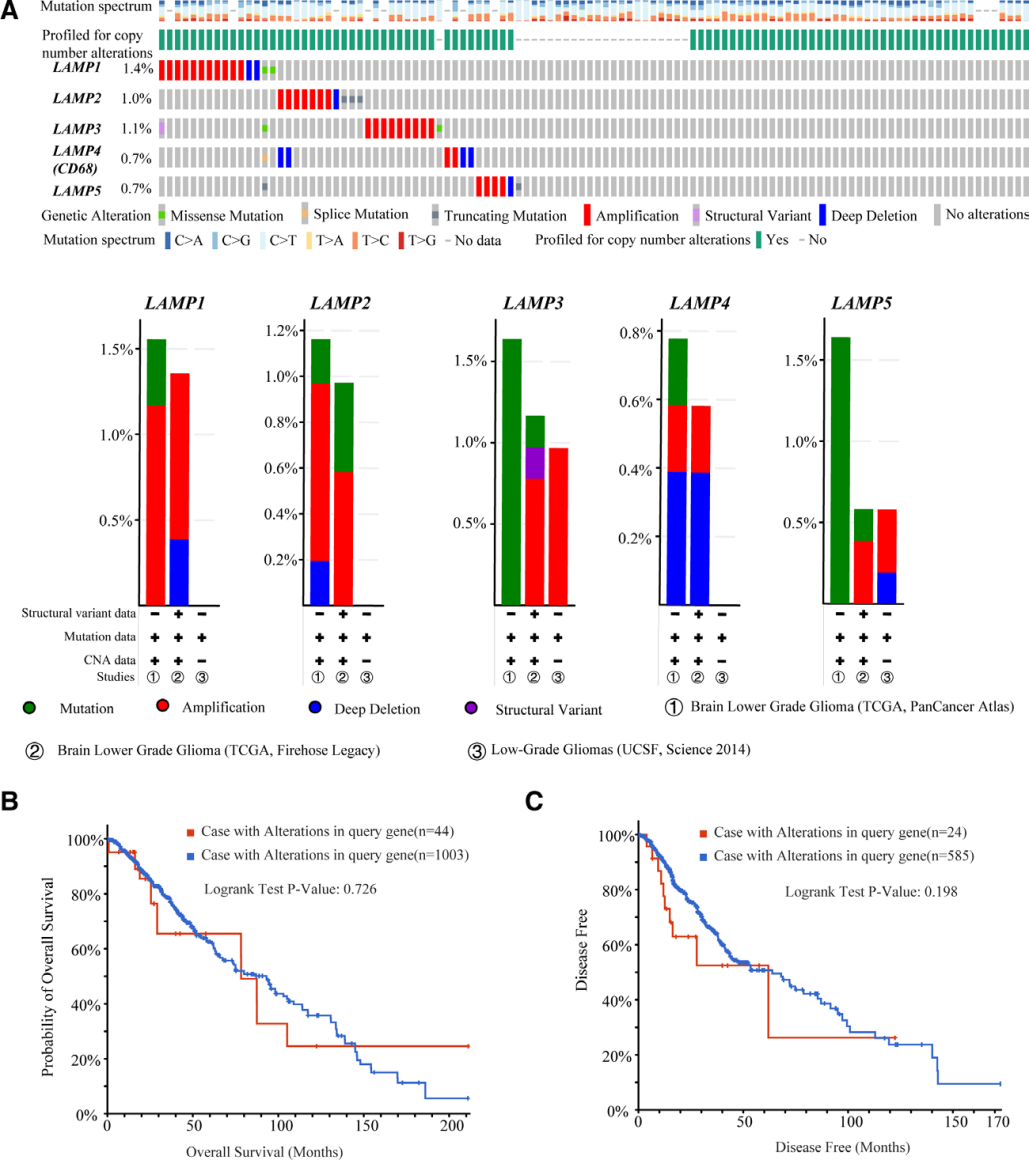
Alteration frequency of LAMPs has no significant correlation with LGG prognosis (TCGA and cBioPortal). (A) OncoPrint visualization of alterations associated with LAMPs genes. (B) Overall survival percentage and survival time of LGG patients with/without gene alterations by Kaplan–Meier plots. (C) Disease free survival percentage and survival time of LGG patients with/without gene alterations by Kaplan–Meier plots, LAMPs = lysosomal associated membrane proteins, LGG = Brain lower grade glioma.

Genetic alteration represents one of the main causes to cancer. To investigate the correlation between LAMP family members genetic alteration with LGG, we used the cBioPortal online tool to collect 3 studies Brain Lower Grade Glioma (TCGA, Pan Cancer Atlas), Brain Lower Grade Glioma (TCGA, Firehose Legacy), Low-Grade Gliomas (UCSF, Science 2014) data of a total of 1105 samples from the TCGA dataset of LGG for analysis. The genetic alteration rate of LAMP1, LAMP2, LAMP3, LAMP4, and LAMP5 was 1.4%, 1.0%, 1.1%, 0.7%, and 0.7%, respectively (Fig. 1A). The results of these studies show that LAMP family members total alteration rate is below 1.4%, these genetic changes include deep deletion, mutation (mis-sense mutation, truncating mutation, and splice mutation). In detail, we calculated the genetic alteration of the LAMP family members in LGG in each individual study, it was show that alteration rate is below 2% in every single study (Fig. 1A). Based on the TCGA provisional dataset, Kaplan–Meier plots were used to evaluate the relationship between LAMP family gene alteration and overall survival and disease-free survival of cases. The result showed no significant difference in the overall survival and disease-free survival between LAMPs gene altered group and unaltered group (Fig. 1B and C).

### 3.2. LAMPs mRNA levels and the prognostic value of the individual LAMPs in LGG

**Figure 2.**
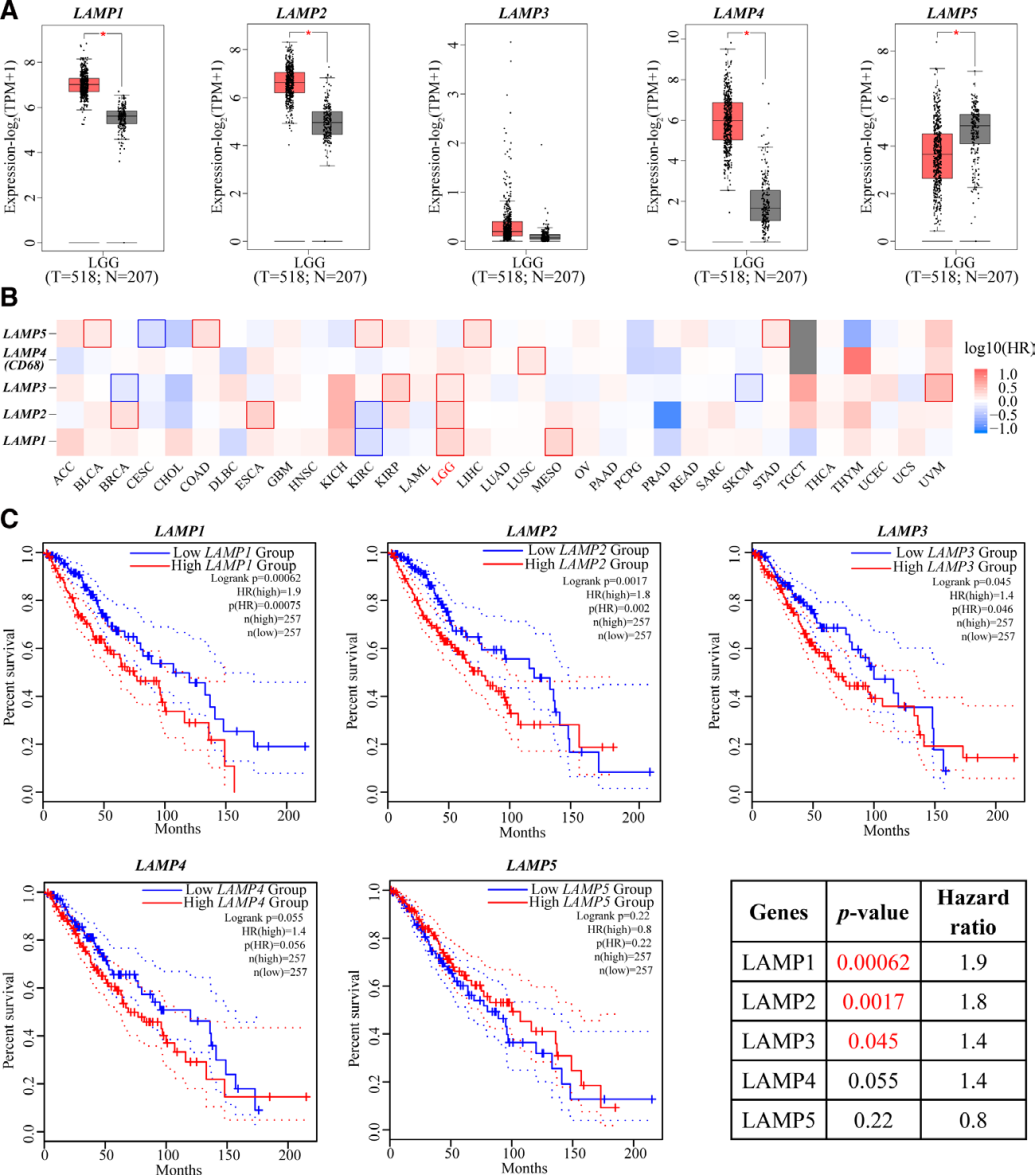
LAMPs mRNA levels and the prognostic value of the individual LAMPs in LGG (GEPIA2 database). (A) The distribution of LAMP1–5 gene mRNA expression between LGG and normal controls. n (tumor, T) = 518; n (normal control, N) = 207. (B) Survival map of Hazardous Ratio of LAMPs in pan cancer, the X-axis is the name of the gene, and the Y-axis is the abbreviation of the name of the cancer. In the heat map, the one selected by the square box indicates that the gene expression level has a significant correlation with the overall survival rate in the corresponding cancer. The red box indicates that the gene expression level has a lower survival rate in the corresponding cancer type. Conversely, the blue box indicates that high expression of the gene has a higher overall survival rate in the corresponding cancer type. (C) Curves show relative expression of LAMP1–5 with overall survival between LGG and normal controls using GEPIA2, n (high) = 257, n (low) = 257. *P* value of log-rank and hazard ratio were listed. LAMP1 = lysosomal associated membrane protein 1, LAMPs = lysosomal associated membrane proteins, LGG = Brain lower grade glioma.

To detect the relationship between mRNA expression profiles of LAMP family members and LGG, we evaluated the expression of LAMP family members from 518 LGG patients and 207 normal controls using the GEPIA2 database. As shown in Figure 2A, the mRNA levels of LAMP1, LAMP2, and LAMP4 showed significantly elevated in LGG patients compared to the normal controls. In addition, we observed that the expression of LAMP5 was significantly down regulation in LGG patients (Fig. 2A). We observed the survival curves of LAMP family members in all cancers, and found that in all types of cancers, the high expression of LAMPs was most correlated with poor prognosis of LGG, among which, the high expression of LAMP1, LAMP2, and LAMP3 genes in LAMPs was significantly correlated with poor prognosis of LGG (Fig. 2B). In detail, the overall survival rates showed significant negatively corelative with high LAMP1, LAMP2, and LAMP3 level through GEPIA2 curve and log-rank test analyses (Fig. 2C).

### 3.3. Association of mRNA expression of different LAMPs with histological subtypes of LGG patients

**Figure 3.**
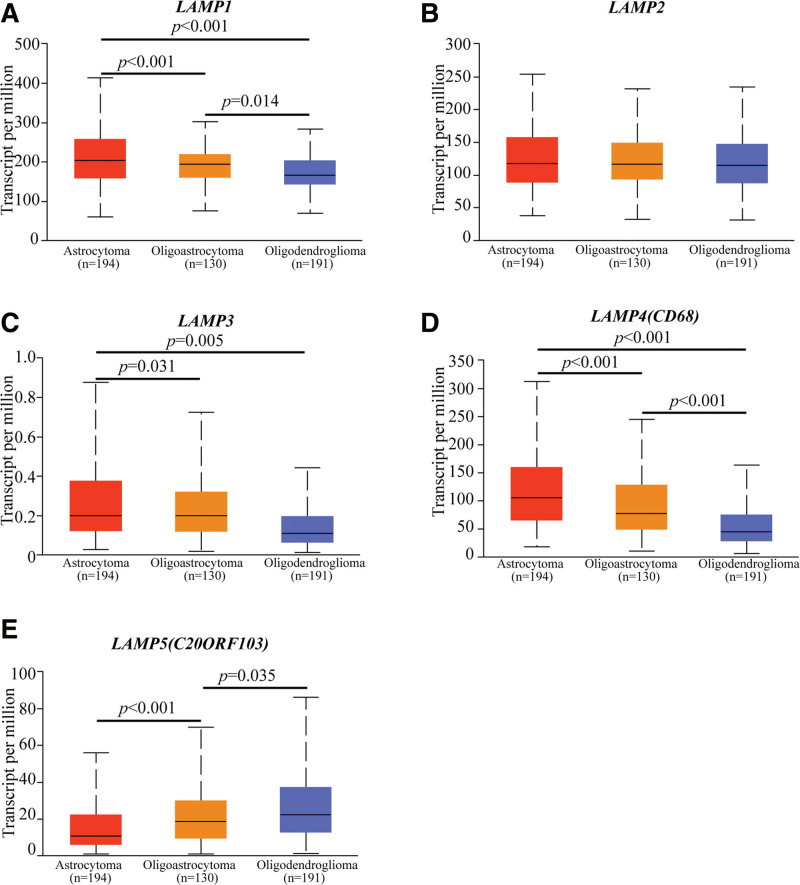
Association of mRNA expression of different LAMPs with histological subtypes of LGG patients (UALCAN database). (A) LAMP1 expression profile based on histological subtypes (Astrocytoma vs oligoastrocytoma, *P* value < .001; Astrocytoma vs Oligodendroglioma, *P* value < .001; Oligoastrocytoma-vs Oligodendroglioma, *P* value < .001). (B) LAMP2 expression profile based on histological subtypes. (C) LAMP3 expression profile based on histological subtypes (Astrocytoma vs oligoastrocytoma, *P* value = .031; Astrocytoma vs Oligodendroglioma, *P* value = .005). (D) LAMP4 expression profile based on histological subtypes (Astrocytoma vs oligoastrocytoma, *P* value < .001; Astrocytoma vs Oligodendroglioma, *P* value < .001; Oligoastrocytoma-vs Oligodendroglioma, *P* value < .001). (E) LAMP5 expression profile based on histological subtypes (Astrocytoma vs oligoastrocytoma, *P* value < .001; Oligoastrocytoma-vs Oligodendroglioma, *P* value = .035). Difference of transcriptional expression was compared by students test and *P* < .05 was considered as statically significant. LAMP1 = lysosomal associated membrane protein 1, LAMP2 = lysosomal associated membrane protein 2, LAMP3 = lysosomal associated membrane protein 3, LAMPs = lysosomal associated membrane proteins, LGG = Brain lower grade glioma.

After mRNA expression and protein expression were found to be over-expressed in LGG patients, we next analyzed the relationship between mRNA expression of different LAMPs family members with histological subtypes of LGG patients by UALCAN (http://ualcan.path.uab.edu), including astrocytoma, oligoastrocytoma and oligodendroglioma. As was shown in Figurer 3, mRNA expressions of LAMP1 and LAMP4 were remarkably correlated with histological subtypes, and patients who were in astrocytoma tended to express higher mRNA expression of LAMPs. In LGG, LAMP1 expression was significantly different among 3 histological subtypes (Astrocytoma vs oligoastrocytoma, *P* value < .001; Astrocytoma vs Oligodendroglioma, *P* value < .001; Oligoastrocytoma-vs Oligodendroglioma, *P* value = .014) (Fig. 3A). LAMP4 expression was significantly different among 3 histological subtypes (Astrocytoma vs oligoastrocytoma, *P* value < .001; Astrocytoma vs Oligodendroglioma, *P* value < .001; Oligoastrocytoma-vs Oligodendroglioma, *P* value < .001) (Fig. 3D). We observed that histological subtypes in LGG was independent of LAMP2 expression (Fig. 3B). In addition, the histological subtypes of LGG were not completely correlated with the expression level of LAMP3 (Astrocytoma vs oligoastrocytoma, *P* value = .031; Astrocytoma vs Oligodendroglioma, *P* value = .005) (Fig. 3C), and the expression level of LAMP5 was correlated with the 2 histological subtypes of LGG (Astrocytoma vs oligoastrocytoma, *P* value < .001; Oligoastrocytoma-vs Oligodendroglioma, *P* value = .035) (Fig. 3E). The *P* values of LAMPs expression and histological subtypes were listed in Table 1, *P* value < .05 are considered significant, and *P* value are marked in red. In short, the results above suggested that mRNA expressions of LAMP1 and LAMP4 were significantly associated with histological subtypes in LGG patients.

**Table 1:** The *P* values of LAMPs expression and histological subtypes.

Gene symbol	Comparison	*P* values
LAMP1	Astrocytoma vs-oligoastrocytoma	<.001
	Astrocytoma-vs-oligodendroglioma	<.001
	Oligoastrocytoma-vs-oligodendroglioma	.014
LAMP2	Astrocytoma-vs-oligoastrocytoma	.36
	Astrocytoma-vs-oligodendroglioma	.28
	Oligoastrocytoma-vs-oligodendroglioma	.91
LAMP3	Astrocytoma-vs-oligoastrocytoma	.031
	Astrocytoma-vs-oligodendroglioma	.005
	Oligoastrocytoma-vs-oligodendroglioma	.18
LAMP4	Astrocytoma-vs-oligoastrocytoma	<.001
	Astrocytoma-vs-oligodendroglioma	<.001
	Oligoastrocytoma-vs-oligodendroglioma	<.001
LAMP5	Astrocytoma-vs-oligoastrocytoma	.073
	Astrocytoma-vs-oligodendroglioma	<.001
	Oligoastrocytoma-vs-oligodendroglioma	.035

LAMP1 = lysosomal associated membrane protein 1, LAMP2 = lysosomal associated membrane protein 2, LAMP3 = lysosomal associated membrane protein 3, LAMPs = lysosomal associated membrane proteins.

### 3.4. The association between LAMPs family expression and immune infiltration in LGG

**Figure 4.**
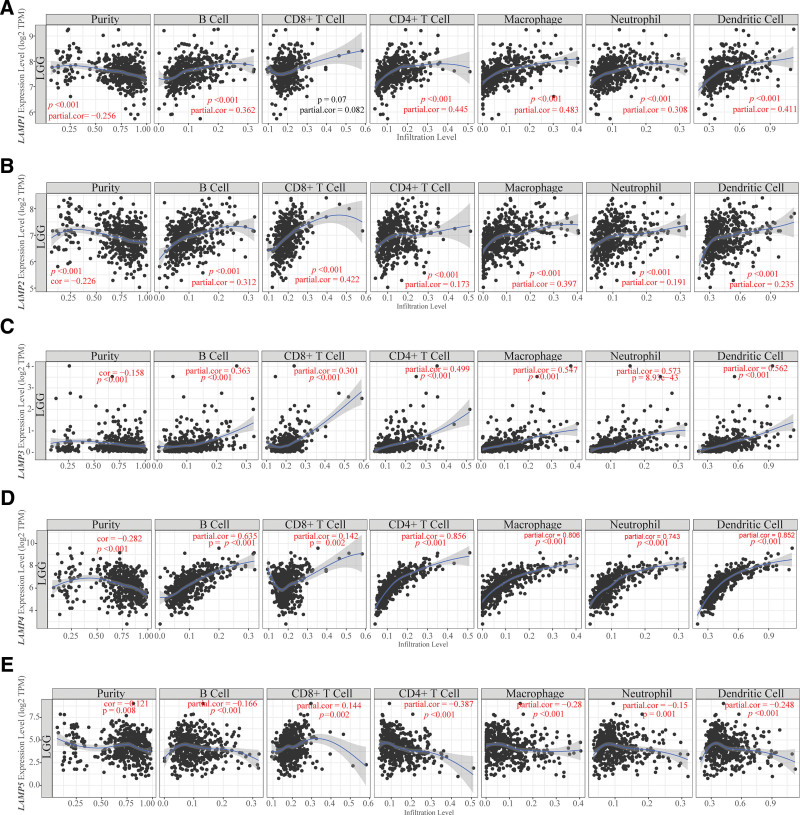
Correlation of LAMPs expression with immune infiltration level in LGG. (A) LAMP1 expression is significantly negatively related to tumor purity and has significant positive correlations with infiltrating levels of B cells, CD4 + T cells, Macrophages, Neutrophils, and Dendritic cells in LGG, other than CD8 + T cells. (B) LAMP2 expression is significantly negatively related to tumor purity and has significant positive correlations with infiltrating levels of B cells, CD8 + T cells, CD4 + T cells, Macrophages, Neutrophils, and Dendritic cells in LGG. (C) LAMP3 expression is significantly negatively related to tumor purity and has significant positive correlations with infiltrating levels of B cells, CD8 + T cells, CD4 + T cells, Macrophages, Neutrophils, and Dendritic cells in LGG. (D) LAMP4 expression is significantly negatively related to tumor purity and has significant positive correlations with infiltrating levels of B cells, CD8 + T cells, CD4 + T cells, Macrophages, Neutrophils, and Dendritic cells in LGG. (E) LAMP5 expression is significantly negatively related to tumor purity, B cells, CD4 + T cells, Macrophages, Neutrophils, Dendritic cells, and has significant positive correlations with infiltrating levels of CD8 + T cells in LGG. LAMP1 = lysosomal associated membrane protein 1, LAMP2 = lysosomal associated membrane protein 2, LAMP3 = lysosomal associated membrane protein 3, LAMPs = lysosomal associated membrane proteins, LGG = Brain lower grade glioma.

TIMER analysis was performed to investigate the relationship between LAMPs expression and tumor-infiltrating lymphocytes in LGG. We were surprised to find that in LGG, the expression of LAMPs (LAMP1–4) was positively correlated with 6 immune cell (B Cell, CD8^+^ T Cell, CD4^+^ T Cell, Macrophage, Neutrophil and Dendritic Cell) infiltration levels in LAMPs except LAMP5(Fig. 4). In detail, B Cell, CD4^+^ T Cell, Macrophage, Neutrophil, Dendritic Cell infiltration levels were found to be positively correlated (purity-corrected partial Spearman rho value > 0, *P* value < .05) with LAMP1 expressions (Fig. 4A). B Cell, CD8^+^ T Cell, CD4^+^ T Cell, Macrophage, Neutrophil and Dendritic Cell infiltration levels were found to be positively correlated with LAMP2 expressions (Fig. 4B). B Cell, CD8^+^ T Cell, CD4^+^ T Cell, Macrophage, Neutrophil and Dendritic Cell infiltration levels were positively correlated with LAMP3 expressions (Fig. 4C). B Cell, CD8^+^ T Cell, CD4^+^ T Cell, Macrophage, Neutrophil and Dendritic Cell infiltration levels were positively correlated with LAMP4 expressions (Fig. 4D). In addition, only CD8^+^ T Cell infiltration levels were positively correlated with LAMP5 expression in LGG, B Cell, CD4 + T Cell, Macrophages, Neutrophils and Dendritic Cell infiltration levels were negatively correlated with LAMP5 expressions in LGG (Fig. 4E). The purity-corrected partial Spearman Rho value and statistical Significance of immune cell infiltration level and the expression of LAMPs in LGG was showing in Table 2, Partial. Cor representative the purity-corrected partial Spearman Rho value and *p* representative statistical Significance, among them, *P* < .05 is red.

**Table 2:** The purity-corrected partial Spearman Rho value and statistical Significance of immune cell infiltration level and the expression of LAMPs in LGG.

Gene	*LAMP1*		*LAMP2*		*LAMP3*		*LAMP4*		*LAMP5*	
*P* value Cell	Partial.cor	*P* value	Partial.cor	*P* value	partial.cor	*P* value	Partial.cor	*P* value	Partial.cor	*P* value
Purity	−0.256	<.001	−0.226	<.001	−0.158	<.001	−0.282	<.001	−0.121	.008
B Cell	0.362	<.001	0.312	<.001	0.363	<.001	0.635	<.001	−0.166	<.001
CD8 + T Cell	0.082	.07	0.422	<.001	0.301	<.001	0.142	<.001	0.144	.002
CD4 + T Cell	0.445	<.001	0.173	<.001	0.499	<.001	0.856	<.001	−0.387	<.001
Macrophage	0.483	<.001	0.397	<.001	0.547	<.001	0.806	<.001	−0.280	<.001
Neutrophil	0.308	<.001	0.191	<.001	0.573	<.001	0.743	<.001	−0.150	.001
Dendritic Cell	0.411	<.001	0.235	<.001	0.562	<.001	0.852	<.001	−0.248	<.001

LAMP1 = lysosomal associated membrane protein 1, LAMP2 = lysosomal associated membrane protein 2, LAMP3 = lysosomal associated membrane protein 3, LAMPs = lysosomal associated membrane proteins, LGG = lower grade glioma.

### 3.5. Expression profiles of LAMPs in TP53 mutant and wild type cases in LGG.

**Figure 5.**
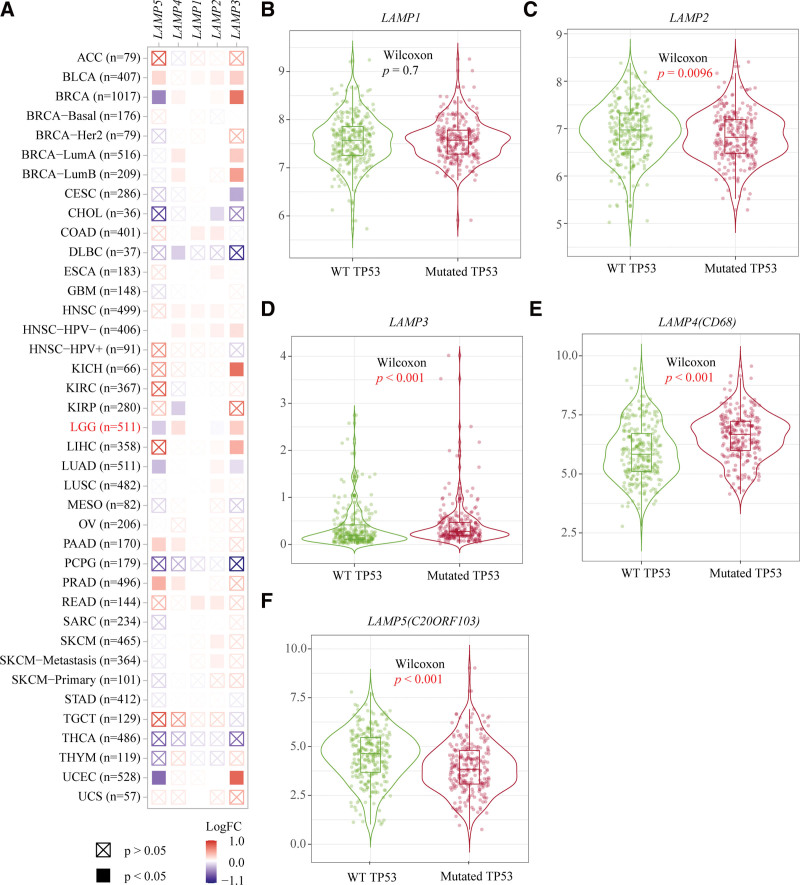
Expression profiles of LAMPs in TP53 mutant and wild type cases in LGG. (A) Heatmap shows the log_2_ fold changes of the differential expression of each gene for each cancer type, red squares represent high expression of LAMPs in LGG of TP53 mutant and blue squares represent low expression of LAMPs in the LGG of TP53 mutant. (B–E) Expression profiles of LAMP1-5 in TP53 mutant and wild type cases in LGG, *P* values are marked in red when *P* value < .05. LAMP1 = lysosomal associated membrane protein 1, LAMPs = lysosomal associated membrane proteins, LGG = Brain lower grade glioma.

We used 511 LGG samples from the TIMER2.0 database to explore the expression levels of LAMPs in TP53 wild type and mutant types in LGG. Heatmap shows the log_2_ fold changes of the differential expression of each gene for each cancer type, we found greater differences in the expression levels of LAMPs between the TP53 mutant and the wild type in LGG in all types of cancer (Fig. 5A). LAMP1 showed no significant difference in the expression level of TP53 mutant and wild type in LGG, *P* = .7 (Fig. 5B). However, LAMP2 (*P* = .0096) and LAMP5 (*P* < .001) were significantly under expressed in samples of TP53 mutant in LGG compared to TP53 wild type (Fig. 5C and F). In addition, LAMP3 (*P* < .001) and LAMP4 (*P* < .001) were significantly overexpressed in samples of TP53 mutant in LGG (Fig. 5D and E).

### 3.6. Genome-wide association of LAMPs mRNA in cancer

**Figure 6.**
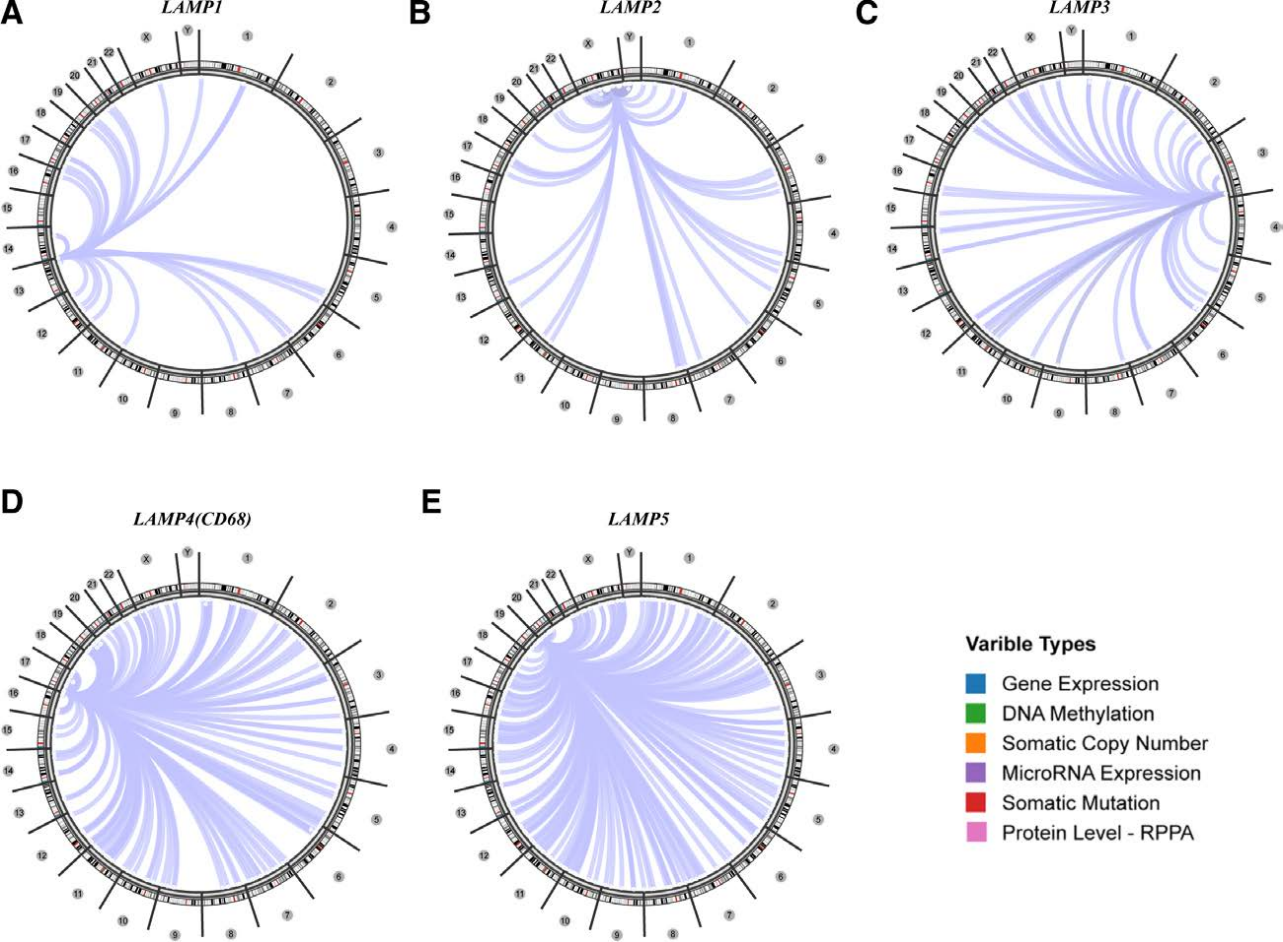
The genome-wide correlation between LAMPs family members and other signatures in LGG from the TCGA database. LAMPs = lysosomal associated membrane proteins, LGG = Brain lower grade glioma.

Using the Regulome Explorer web tool, we further explored the relevant genomic correlations between certain signatures and LAMPs in LGG. Based on the associations among gene, deoxyribonucleic acid (DNA) methylation, somatic copy number, somatic mutation and protein level, circus plots were displayed to illustrate these interrelations in human cancers. According to the data from TCGA, associations could be detected between LAMPs and other signatures in LGG (Fig. 6). Detailed data can be found in Supplemental Digital Content, http://links.lww.com/MD/J452. We found that LAMP1 to 3 has 30 to 100 genes associated with LGG.

### 3.7. Enrichment analysis and hub gene with LAMP1/2/3 related gene in LGG.

**Figure 7.**
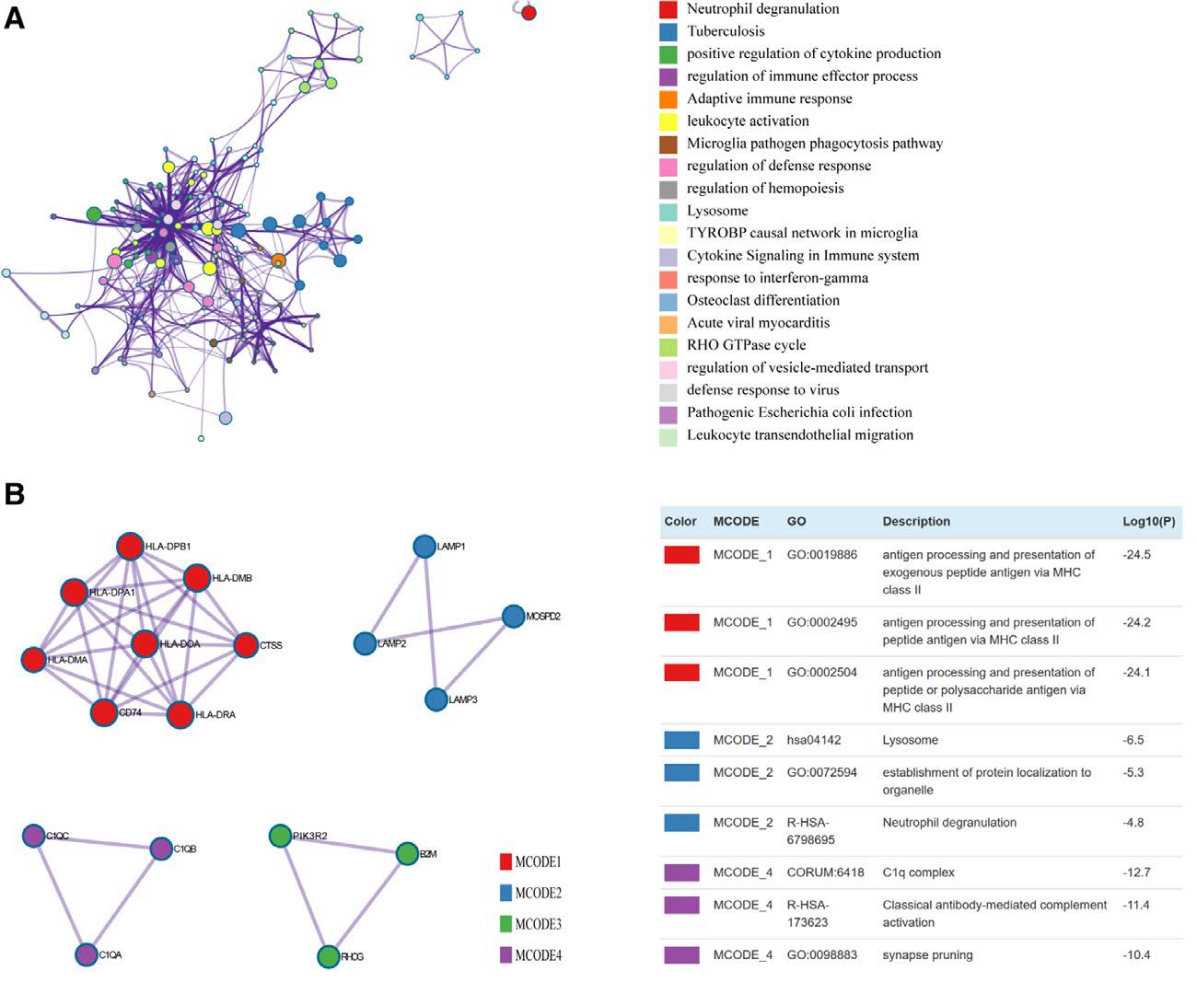
Enrichment analysis with LAMP1\2\3 association gene in LGG. (A) Colored by cluster ID, where nodes that share the same cluster ID are typically close to each other. (B) MCODE components identified in the gene lists (For each given gene list, protein-protein interaction enrichment analysis has been carried out with the following databases: STRING, BioGrid7 OmniPath, InWeb_IM. LAMP1 = lysosomal associated membrane protein 1, LGG = Brain lower grade glioma.

To explore the interactions and internal mechanisms of association genes of LAMP1/2/3, we used Metascape to perform the enrichment analysis, and MCODE analysis of LAMP1/2/3 and their association genes. To capture the relationships between the terms, a subset of enriched terms has been selected and rendered as a network plot, where terms with a similarity > 0.3 are connected by edges. We select the terms with the best *P* values from each of the 20 clusters, with the constraint that there are no more than 15 terms per cluster and no more than 250 terms in total. The network is visualized using Cytoscape5, where each node represents an enriched term and is colored first by its cluster ID. We found that LAMP1/2/3 association genes are mainly enriched in Neutrophil degranulation, Tuberculosis, positive regulation of cytokine production and other pathways and terms (Fig. 7A). MCODE components identified in the gene lists (For each given gene list, protein-protein interaction enrichment analysis has been carried out with the following databases: STRING, BioGrid7 OmniPath, InWeb_IM. Only physical interactions in STRING (physical score > 0.132) and BioGrid are used. These MCODE include antigen processing and presentation of exogenous peptide antigen via MHC Class II, 9) establishment of protein localization to organelle, classical antibody-mediated complement activation (Fig. 7B). Importantly, enrichment analysis applied to each MCODE component indicated that biological function was primarily associated with series of pathways in synapse and immunity.

## 4. Discussion

In this study, we collected several sets of data from multiple databases including GEPIA2, cBioPortal, UALCAN and TIMER performed comprehensive bioinformatic analysis to evaluate the role of LAMPs in LGG. We did not find significant correlation between LAMPs gene mutation and LGG prognosis. However, the LAMP1/2/4 mRNA levels were significantly higher in LGG patients than in healthy controls. Moreover, high mRNA expressions of LAMP1/2/3 were associated with poor overall survival.

LAMP1 is a Protein Coding gene. Among its related pathways are Cytoskeletal Signaling and Senescence and autophagy in cancer.^[25,26]^ LAMP1 is involved in the malignant progression of multiple human cancers, such as head and neck squamous cell carcinoma,^[27]^ sckin cutaneous melanoma,^[28]^ breast carcinoma,^[29]^ colon adenocarcinoma,^[30]^ epithelial ovarian cancer,^[31]^ even gliomas.^[14,32]^ There are statistics that suggest high LAMP1 expression is associated with unfavorable prognosis in laryngeal squamous cell carcinoma patients.^[33]^ Krassimir Dangalov used immunohistochemistry and qPCR to detect the protein and mRNA levels of LAMP1 and LAMP2 in newly diagnosed high-grade gliomas patients and healthy controls. The data showed that LAMP1 overexpression in high-grade gliomas are presented suggesting involvement of this gene and protein in cell adhesion and tumor progression,^[34]^ these findings might help the elucidation of the complex biological role of the multifunctional LAMPs proteins and to predict novel therapeutic targets in lysosomes. Database from TCGA show that LAMP1 alteration rate is 1.4%, it is suggested that LAMP1 has a small mutation probability in LGG and has little genetic effect. LAMP1 were found significantly overexpressed in LGG. Importantly, high LAMP1 expression was significantly associated with poor overall survival in LGG patients. In addition, mRNA expressions of LAMP1 were remarkably correlated with histological subtypes, and patients who were in astrocytoma tended to express higher mRNA expression of LAMP1. At the same time, B Cell, CD4 + T Cell, Macrophage, Neutrophil, Dendritic Cell infiltration levels were found to be positively correlated with LAMP1 expression. LAMP2 is a member of a family of membrane glycoproteins. LAMP2 plays an important role in chaperone-mediated autophagy.^[35,36]^ It may play a role in tumor cell adhesion.^[37]^ And have function in the protection, maintenance, and adhesion of the lysosome. High expression of LAMP2 has been reported to promote tumor development in multiple human cancers, including esophageal squamous cell carcinoma and hepatocellular carcinoma.^[38–41]^ Our data from GEPIA2 database showed that LAMP2 were found significantly overexpressed in LGG, and high LAMP2 expression was significantly associated with poor overall survival in LGG patients.

We used the Regulome Explorer web tool, we further explored the relevant genomic correlations between certain signatures and LAMPs in LGG. We employed Metascape database to perform enrichment analysis of LAMP1/2/3 genomic correlated genes. The results of the functional analysis of distinct LAMP and genomic association genes indicated that these genes were involved in multiple pathways related to immune system and nervous system, such as neutrophil degranulation, positive regulation of cytokine production, Microglia pathogen phagocytosis pathway and tyrobp causal network in microglia. At the same time, our data showed that the results of immune infiltration were also significant, and we guessed that LAMP1/2/3 might affect the progress of LGG through immunity and autophagy, the results of our immune infiltrates analysis at LGG strongly support this view.

## 5. Conclusion

This study conducted a comprehensive assessment of the prognostic relevance of LAMPs in LGG via multiple bioinformatics analysis and suggest that LAMP1/2/4 is a potential diagnostic marker for LGG, and LAMP1/2/3 is a potential prognostic marker for LGG. In conclusion, LAMP1/2 as diagnostic and prognostic marker for LGG.

## Author contributions

**Data curation:** Xiao Fen Qiu.

**Funding acquisition:** Xiaoli Chen.

**Investigation:** Xiaoli Chen.

**Supervision:** Xiaoli Chen.

**Visualization:** Xiao Fen Qiu.

**Writing – original draft:** Xiao Fen Qiu.

## Supplementary Material


